# An Efficient, Anonymous and Robust Authentication Scheme for Smart Home Environments

**DOI:** 10.3390/s20041215

**Published:** 2020-02-22

**Authors:** Soumya Banerjee, Vanga Odelu, Ashok Kumar Das, Samiran Chattopadhyay, Youngho Park

**Affiliations:** 1 Department of Information Technology, Jadavpur University, Salt Lake City, Kolkata 700 098, India; soumyaBanerjee@outlook.in (S.B.); samirancju@gmail.com (S.C.); 2 Department of Computer Science and Information Systems, Birla Institute of Technology & Science, Pilani Hyderabad Campus, Hyderabad 500 078, India; odelu.vanga@hyderabad.bits-pilani.ac.in; 3 Center for Security, Theory and Algorithmic Research, International Institute of Information Technology, Hyderabad 500 032, India; iitkgp.akdas@gmail.com or; 4 Northumbria University, Newcastle upon Tyne NE1 8ST, UK; 5 School of Electronics Engineering, Kyungpook National University, 80 Daehak-ro, Sangyeok-dong, Buk-gu, Daegu 41566, Korea

**Keywords:** Internet of Things (IoT), smart homes, anonymous authentication, session key agreement, security, Automated Validation of Internet Security Protocols and Applications (AVISPA)

## Abstract

In recent years, the Internet of Things (IoT) has exploded in popularity. The smart home, as an important facet of IoT, has gained its focus for smart intelligent systems. As users communicate with smart devices over an insecure communication medium, the sensitive information exchanged among them becomes vulnerable to an adversary. Thus, there is a great thrust in developing an anonymous authentication scheme to provide secure communication for smart home environments. Most recently, an anonymous authentication scheme for smart home environments with provable security has been proposed in the literature. In this paper, we analyze the recent scheme to highlight its several vulnerabilities. We then address the security drawbacks and present a more secure and robust authentication scheme that overcomes the drawbacks found in the analyzed scheme, while incorporating its advantages too. Finally, through a detailed comparative study, we demonstrate that the proposed scheme provides significantly better security and more functionality features with comparable communication and computational overheads with similar schemes.

## 1. Introduction

Interest in the Internet of Things (IoT) has grown exponentially over recent years, and it is likely to continue growing for the foreseeable future [1]. The smart home as an important IoT application has also gained much interest in recent years. Adoption of home automation systems for monitoring and controlling various smart devices is at an all-time high [2,3]. The reduced operating expenses, coupled with the increased quality of life, encourage the users to rely on these more and more. A smart home reduces expenses while providing higher comfort, security and safety to the users [4]. Additionally, smart homes can provide the elderly and disabled with prompt medical care based on the readings of smart gadgets [5]. However, as a direct result of using these services, a large volume of private and sensitive data is being transmitted over insecure networks. Security and privacy are considered the fundamental requirements for consumer technology deployment [6].

Consider a smart gadget for monitoring a patient. In order to get medical services, the external user (for example, a doctor) needs to have direct access to data sensed by the sensors in the gadget monitoring the patient’s body. Such information will invariably include current vital readings like blood sugar level, blood pressure, etc. For obvious reasons, this information needs to private and confidential. Similarly, data generated from the surveillance system, temperature and movement sensors, or control data for lighting or other appliances need to be secure and confidential. Devices in a smart home can be accessed through a gateway node that connects them to the Internet. To ensure data privacy and integrity, various entities, such as the users, the smart devices, and the gateway node need to generate session keys after their mutual authentication. The generated session keys can then be used for further communication without fear of data compromise.

### 1.1. Network and Threat Models

**Figure 1:**
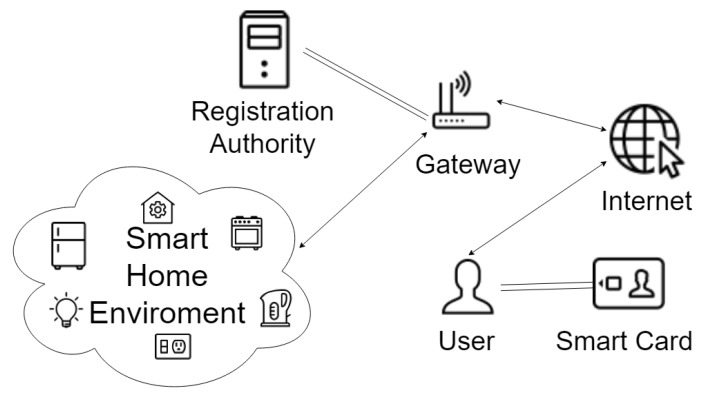
A typical smart home architecture (adapted from [7]).

We follow the widely accepted network model for the proposed scheme, which is defined in the typical smart home architecture [7] shown in Figure 1. The smart devices connect to the public Internet through the gateway nodes (GWN). Users (*U*) and smart devices (SD) must be registered or enrolled with the registration authority RA before operating in the network. The RA is a fully trusted entity in the network. The registered mobile users can avail of the services provided by the already enrolled smart devices through the gateway node and negotiate the session keys after mutual authentication.

We evaluate the proposed scheme under the de-facto standard “Dolev-Yao (DY) threat model” [8]. In the DY-threat model, an adversary, say A, has ultimate authority over the communication channel, and consequently he/she is capable of eavesdropping, modifying, dropping, or even inserting forged messages for any communicated messages. Furthermore, it is assumed that A can physically capture some smart devices, as monitoring the devices 24/7 is not possible, to extract the sensitive information stored in them using power analysis attacks [9]. Moreover, the smart card of a user can be lost or stolen, and the adversary A can also extract all the sensitive information stored in its memory using power analysis attacks [9]. Both the registration authority (RA) and the gateway node (GWN) are considered trusted in the smart home environment. Furthermore, we use the stronger threat model, known as the “Canetti and Krawczyk’s (CK) adversary model” [10], wherein the adversary A, in addition to having all capacities of the DY-therat model, can also compromise ephemeral information like session-specific states and keys. Thus, in the presence of the CK-adversary, a user authentication scheme must be designed such that leakage of ephemeral secrets should have minimal impact on the security of unrelated entities in the authenticated key-exchange scheme [11].

### 1.2. Research Contributions

The main contributions are given below.

- We first analyze the recently proposed anonymous authentication scheme by Shuai et al. [7] for the smart home environment and then highlight that their scheme fails to resist known attacks, such as privileged-insider attack, through offline password guessing and lost/stolen smart card attacks, user impersonation attacks, parallel session attacks, and password change attacks.
- We present a more secure user authentication scheme that avoids the security pitfalls demonstrated in Shuai et al.’s scheme.
- Through formal as well as informal security analysis, we show the resistance of the proposed scheme against various potential attacks needed in a smart home environment.
- We then present a comparative study to demonstrate the superior security and functionality features of the proposed scheme relative to the existing relevant authentication schemes.
- Finally, we provide a practical perspective on the applicability of the proposed scheme through a network simulator (NS3) simulation study.

### 1.3. Related Work

In the last decade, several authors investigated the issues of remote authentication for smart homes. Jeong et al. [12] suggested an authentication protocol for home networks based on “One-Time Passwords (OTPs)” and smart cards. However, their scheme not only transmitted the user identities in plaintext, but also did not provide mutual authentication. Vaidya et al. [13] designed a “remote authentication scheme using lightweight computation modules”. Unfortunately, Kim et al. demonstrated that [13] was not only vulnerable to known attacks, but it also failed to provide “user anonymity” and “forward secrecy”. To strengthen the security, Kim et al. presented an improved scheme [14] over the Vaidya et al. scheme. [13].

Vaidya et al. [15] presented an “Elliptic Curve Cryptography (ECC)” based device authentication scheme for smart home networks. However, their scheme was found to be susceptible to privileged-insider, password guessing, and user impersonation attacks. Pradeep and Singh [16] proposed a secure three-factor authentication scheme for “ubiquitous computing devices” with a pass-phrase based device integrity check.

Li proposed a lightweight key establishment scheme [17] as a solution to the security issue in smart home energy management systems. Unfortunately, their scheme was not scalable as it requires the management of many keys and certificates. Around the same time, Han et al. [18] designed a key agreement scheme for a secure pairing process for smart home systems. But, their scheme depends on an always-online service by the manufacturer of the devices, which is an infeasible requirement. Additionally, neither the scheme [17] nor the scheme [18] provided “mutual authentication between user and smart devices”.

Santoso and Vun [19] suggested an “ECC -based authentication scheme for smart homes”, where they presented the idea of using the Wi-Fi gateway as the central node of the system. Unfortunately, their scheme was vulnerable to privileged–insider attack, and consequently, it failed to guarantee user anonymity and untraceability properties.

Kumar et al. [4] designed a “lightweight anonymity preserving authentication scheme for smart home environments”. However, their scheme failed to provide “mutual authentication between the user and the smart device”. In their scheme, user anonymity and untraceability properties are also compromised.

Wazid et al. [20] suggested a lightweight remote user authentication scheme for the smart home environment which fulfills the design criteria for the smart home environment. Yu and Li [21] proposed another user authentication scheme for the smart home environment. However, their protocol did not necessitate a secure environment for user and device registration. Moreover, their scheme relied on bilinear pairing operations, and as a result, their scheme incurs exceptionally high overheads. Shuai et al. [7] designed an “ECC-based authentication scheme for the smart home environment”. However, in this paper, we discuss the advantages and limitations of their scheme in detail. Naoui et al. [22], Fakroon et al. [23] and Dey and Hossain [24] also presented other user authentication schemes for the smart home environment.

## 2. Review of Shuai et al.’s Scheme

In this section, we briefly review Shuai et al.’s scheme. Their scheme has the following phases: (a) initialization phase, (b) registration phase, (c) login and authentication phase, and (d) password change phase. In this section, we only review the first three phases, and the details regarding the password change phase can be found in the scheme [7].

### 2.1. Initialization Phase

During initialization, the registration authority (RA) selects an elliptic curve $E(F_{p})$ of the form $y^{2}=x^{3}+ax+b\;\left(\mathrm{mod}\;p\right)$ of order *p* over finite field $F_{p}$ with a generator point *P*, where *p* is a large prime number and $a,b\in Z_{p}=\left\{0,1,\cdots ,p-1\right\}$ such that $4a^{3}+27b^{2}\neq 0\;\left(\mathrm{mod}\;p\right)$. RA then creates a private key *x* and calculates the corresponding public key X=x·P. RA selects a “long term key *K*” and a “cryptographic one-way collision-resistant hash function $h{\left(\cdot \right)}^{*}:{\left\{0,1\right\}}^{*}\to Z_{p}^{*}$”, where $Z_{p}^{*}=\left\{1,2,\cdots ,p-1\right\}$. RA commits *x* and *K* to the GWN and makes $\left\{E\left(F_{p}\right),P,X,h(\cdot )\right\}$ public. RA also picks and saves GID into gateway node’s memory as its unique identity. In addition, RA generates $SID_{d}$ as a random unique identity for each smart device SD. These identities are saved to the respective smart devices SD.

### 2.2. Registration Phase

This phase comprises of the user registration as well as the smart device enrollment phases.

#### 2.2.1. User Registration

A user *U* registers with the RA through the following steps:

- **Step 1.***U* first picks his/her identity $ID_{u}$, password $PW_{u}$ and generates a random secret *a*. *U* then calculates pseudo-password $HPW_{u}=h(PW_{i}\|a)$ and securely dispatches the credentials $\left\{ID_{u},HPW_{u}\right\}$ to RA.
- **Step 2.** If $ID_{u}$ is already registered, RA rejects the request. Otherwise, RA computes $K_{UG}=h(ID_{u}\|K)$, $A_{1}=K_{UG}⊕HPW_{u}$. RA generates a random value TEMP in order to record the number of user login failures, and sets TEMP=0. Next, RA writes $\left\{A_{i},TEMP\right\}$ to a smart card $SC_{u}$ and securely issues $SC_{u}$ to the user *U*.
- **Step 3.** On receiving the smart card $SC_{u}$, *U* calculates $A_{2}=a⊕h(ID_{u}\|PW_{u})$ and $A_{3}=h(ID_{u}\|HPW_{u})$, and appends $A_{2}$ and $A_{3}$ to the smart card $SC_{u}$. The smart card $SC_{u}$ finally contains the credentials $\left\{A_{1},A_{2},A_{3},TEMP\right\}$.

#### 2.2.2. Device Enrollment

The steps for smart device, SD’s enrollment with the RA:

- **Step 1.**SD first securely transmits its identity $SID_{d}$ to RA.
- **Step 2.** If SD is already enrolled, the request is rejected by the RA. Otherwise, RA computes $K_{GS}=$$h(SID_{d}\|K)$ and securely sends the secret key $K_{GS}$ to SD.
- **Step 3.** On receiving the reply, SD saves the secret key $K_{GS}$ in its memory.

### 2.3. Login and Authentication Phase

For a registered user *U* to access a smart device SD, he/she must first establish a session key SK after“ mutual authentication between *U*, SD and GWN”. The steps for login, and authentication and session key establishment phase are as follows:

- **Step 1.** User *U* first enters his/her identity $ID_{u}$ and password $PW_{u}$, and calculates $a^{*}=A_{2}⊕h(ID_{u}\|PW_{u})$, $HPW_{u}^{*}=h(PW_{u}\|a^{*})$ and $A_{3}^{*}=h(ID_{u}\|HPW_{u}^{*})$. Only if the check $A_{3}^{*}=A_{3}$ holds, the login is successful. In case of a failed login attempt, the smart card $SC_{u}$ of the user *U* updates TEMP=TEMP+1. This value records the login attempts and if it exceeds a pre-defied threshold, the user *U* is considered as compromised and is suspended till he/she re-registers.
  After a successful login, the smart card $SC_{u}$ generates two random numbers $R_{1}$ and $w\in Z_{p}^{*}$, and computes $K_{GU}=$$A_{1}⊕HPW_{u}$, $A_{4}=w\cdot P$, $A_{5}=w\cdot X$, $DID_{u}=ID_{u}⊕A_{5}$, $M_{1}=$$(R_{1}\|SID_{d})⊕K_{UG}$ and $V_{1}=$$h(ID_{u}\|R_{1}\|K_{UG}\|M_{1})$, and sends the login request message $\langle DID_{u},$$A_{4},$$M_{1}$$V_{1}$〉 to GWN via open channel.
- **Step 2.** On receiving the login request $\langle DID_{u},$$A_{4},$$M_{1}$$V_{1}$〉, GWN computes $A_{5}^{*}=x\cdot A_{4}$, $ID_{u}^{*}=DID_{u}⊕A_{5}^{*}$, $K_{GU}=h(ID_{u}^{*}\left\|K\right)$, $(R_{1}^{*}\|SID_{d})=M_{1}⊕K_{GU}$, $V_{1}^{*}$$=h(IDi^{*}\|R1^{*}\|K_{GU}\|M_{1})$. Only if the condition $V_{1}^{*}=V_{1}$ holds, GWN believes the legitimacy of the login request. GWN then generates a random number $R_{2}\in Z_{p}^{*}$ and computes $K_{GS}=h(SID_{d}\left\|K\right)$, $M_{2}$$=(ID_{u}\left\|GID\right\|R_{1}\|R_{2})⊕K_{GS}$, $V_{2}$$=h(ID_{u}\|GID\|K_{GS}\|R_{1}\|R_{2})$. Finally, GWN sends the authentication request message $\langle M_{2}$, $V_{2}\rangle$ to SD via public channel.
- **Step 3.** On receiving the message $\langle M_{2}$, $V_{2}\rangle$, SD calculates $(ID_{u}\left\|GID\right\|R_{1}\|R_{2})=M_{2}⊕K_{GS}$, $V_{2}^{*}=h(ID_{u}\left\|GID\right\|$$K_{GS}\|R_{1}\|R_{2})$ and checks if $V_{2}^{*}=V_{2}$. If true, SD generates a random number $R_{3}\in Z_{p}^{*}$ and computes $SK=h(ID_{u}\|GID\|SID_{d}\|R_{1}\|R_{2}\|R_{3})$, $M_{3}$$=R_{3}⊕K_{GS}$, $V_{3}=h(R_{3}\|K_{GS}\left\|SK\right)$ and finally transmits the authentication reply message $\langle M_{3},V_{3}\rangle$ to GWN.
- **Step 4.** On receiving the message $\langle M_{3},V_{3}\rangle$ from SD, GWN computes $R_{3}=M_{3}⊕K_{GS}$, $SK=h(ID_{u}\|$$GID\|SID_{d}\|R_{1}\|R_{2}\|R_{3})$, $V_{3}^{*}=h(R_{3}\|K_{GS}\left\|SK\right)$, and if $V_{3}^{*}==V_{3}$, GWN computes $M_{4}=\left(GID\right\|R_{2}\|R_{3})⊕K_{GU}$ and $V_{4}=h(K_{GU}\left\|SK\right\|R_{2}\|R_{3})$, and sends the acknowledgement message $\langle M_{4},V_{4}\rangle$ to *U* via public channel.
- **Step 5.** On receiving the message $\langle M_{4},V_{4}\rangle$ from GWN, *U* computes $\left(GID\right\|R_{2}\|R_{3})=M4⊕K_{GU}$, SK$=h(ID_{u}\|GID\|$$SID_{d}\|R_{1}\|R_{2}\|R_{3})$ and $V_{4}^{*}=h(K_{GU}\left\|SK\right\|R_{2}\|R_{3})$, and if $V_{4}^{*}=V_{4}$, SD is authenticated by the GWN, and also the session key SK is established between *U* and SD.

## 3. Security Vulnerabilities in Shuai et al.’s Scheme

In this section, we cryptanalyze the scheme proposed by Shuai et al. and observe that in the presence of a passive/active adversary, it is vulnerable to several potential attacks. We detail the possible attacks below.

### 3.1. Privileged-insider Attack through Offline Password Guessing and Lost/Stolen Smart Card Attacks

Suppose an adversary A, who is also a privileged insider user, acts as an adversary, say A. In this case, A knows the credentials $ID_{u}$ and $HPW_{u}$ of a legitimate registered user *U* which are submitted to the RA during the user registration phase (see Section 2.2.1), where $HPW_{u}=h(PW_{i}\left\|a\right)$ and *a* is a random secret. Moreover, if A can acquire the lost/stolen smart card $SC_{u}$ of the user *U*, using the “power analysis attacks” [9,25], the adversary A can extract all the credentials $\left\{A_{1},A_{2},A_{3},TEMP\right\}$ stored in the memory of $SC_{u}$, where $K_{UG}=h(ID_{u}\left\|K\right)$, $A_{1}=K_{UG}⊕HPW_{u}$, $A_{2}=a⊕h(ID_{u}\|PW_{u})$ and $A_{3}=h(ID_{u}\|HPW_{u})$. Now, as $A_{2}=a⊕h(ID_{u}\|PW_{u})$ and $HPW_{u}=h(PW_{u}\left\|a\right)$, A can form the following relation:

(1) $$HPW_{u}=h(PW_{u}\|(A_{2}⊕h(ID_{u}\|PW_{u}))).$$

A can then guess a password, say $PW_{u}^{\prime }$. Using the guessed password $PW_{u}^{\prime }$, and $ID_{u}$ and $A_{2}$, A further can calculate $HPW_{u}^{\prime }=$$h(PW_{u}^{\prime }\|$$(A_{2}⊕$$h(ID_{u}$$\|PW_{u}^{\prime })))$, and verify if the condition $HPW_{u}^{\prime }=$$HPW_{u}$ is valid or not. If the condition holds, it means that A is successful in guessing the user *U*’s correct password. Hence, it is clear that the low-entropy guessed passwords are easily guessed and verified in Shuai et al.’s scheme. As a result, Shuai et al.’s scheme is vulnerable to privileged-insider attack with the help of both offline password guessing and lost/stolen smart card attacks.

### 3.2. User Impersonation and Parallel Session Attacks

A privileged insider adversary A with the knowledge of registration information $ID_{u}$ and $HPW_{u}$, and extracted $A_{1}$ from the stolen smart card $SC_{u}$ of a valid registered user *U* (discussed in Section 3.1) can easily compute secret key $K_{GU}=$$A_{1}⊕HPW_{u}$. Consequently, A can forge the login request message $\langle DID_{u},$$A_{4},$$M_{1},$$V_{1}\rangle$ to the GWN in order to impersonate the user *U* due to the following reason. Since each smart device SD sends its identity $SID_{d}$ to the RA, the privileged insider adversary A of the RA also knows it. Now, A can generate two random numbers $R_{1}^{\prime }$ and $w^{\prime }\in Z_{p}^{*}$, and compute $A_{4}^{\prime }=w^{\prime }\cdot P$, $A_{5}^{\prime }=w^{\prime }\cdot X$, $DID_{u}^{\prime }=ID_{u}⊕A_{5}^{\prime }$, $M_{1}^{\prime }=$$(R_{1}^{\prime }\|SID_{d})⊕K_{UG}$, $V_{1}^{\prime }=$$h(ID_{u}\|R_{1}^{\prime }\|K_{UG}\|M_{1}^{\prime })$. As a result, the adversary A is able to send a valid login request message $\langle DID_{u}^{\prime },$$A_{4}^{\prime },$$M_{1}^{\prime },$$V_{1}^{\prime }\rangle$ to the GWN. Thus, a privileged adversary can impersonate a legal registered user *U* in Shuai et al.’s scheme.

We consider another attack, where privileged insider adversary A of the RA, who has calculated $K_{GU}$ from the previous attack, can intercept the message $\langle M_{4},V_{4}\rangle$ that is sent from the GWN to a user *U*. A, having the knowledge of $K_{UG}$ and $ID_{U}$, can calculate $(GID\|R_{2}\|R_{3})=$$M4⊕K_{GU}$ and the session key SK$=h(ID_{u}$‖GID$\|SID_{d}$$\|R_{1}$$\|R_{2}$$\|R_{3})$. Thus, A can independently calculate the session key SK making the scheme of Shuai et al. vulnerable to the parallel session attack.

### 3.3. Password Change Attack

Suppose a privileged insider of the RA being an adversary A after learning the password $PW_{u}$ from the previously discussed attack in Section 3.1 can simply execute the password update phase to change a legal registered user *U*’s password if the smart card $SC_{u}$ of *U* is being stolen by A. For this purpose, A has the credentials $\{A_{1},$$A_{2},$$A_{3},$TEMP} stored in the memory of $SC_{u}$, where $K_{UG}=h(ID_{u}\left\|K\right)$, $A_{1}=K_{UG}⊕HPW_{u}$, $A_{2}=a⊕h(ID_{u}\|PW_{u})$ and $A_{3}=h(ID_{u}\|HPW_{u})$. A first calculates $K_{GU}=$$A_{1}⊕HPW_{u}$ using previous registration information $HPW_{u}$ and a=$A_{2}⊕h(ID_{u}$$\|PW_{u})$. Next, A chooses his/her own password, say $PW_{u}^{\prime }$ and calculates $HPW_{u}^{\prime }=$$h(PW_{u}^{\prime }$‖a), $A_{1}^{\prime }=$$K_{UG}⊕$$HPW_{u}^{\prime }$, $A_{2}^{\prime }=a⊕h(ID_{u}$$\|PW_{u}^{\prime })$ and $A_{3}^{\prime }=$$h(ID_{u}$$\|HPW_{u}^{\prime })$. Finally, A updates the old credentials $\left\{A_{1},A_{2},A_{3},TEMP\right\}$ with the newly computed credentials $\left\{A_{1}^{\prime },A_{2}^{\prime },A_{3}^{\prime },TEMP\right\}$ in the memory of the smart card $SC_{u}$. This clearly shows that the password change attack is easily mounted on Shuai et al.’s scheme.

## 4. The Proposed Scheme

In this section, we present a more secure “anonymous authentication and session key establishment scheme” for smart home environments, which is free from all the mentioned security vulnerabilities discussed in Section 3. The important phases of our scheme are discussed below.

### 4.1. Initialization Phase

This phase is similar to that presented in Section 2.1. Note that during initialization, the registration authority (RA) also generates a “long term key *K*” and a “ collision-resistant cryptographic one-way hash function $h{\left(\cdot \right)}^{*}:{\left\{0,1\right\}}^{*}\to Z_{p}^{*}$”. RA then commits *K* to GWN and makes {h(·)} public.

### 4.2. Registration Phase

The registration phase details the procedure for dynamic device enrollment and user registration.

#### 4.2.1. Dynamic Device Enrollment

Any time after initialization, a smart device SD can be enrollment with the RA via secure channel through the following steps:

- **Step 1.**SD first securely transmits its identity $SID_{d}$ to RA.
- **Step 2.** If SD is already enrolled, the request is rejected by the RA. Otherwise, RA computes the secret key $K_{GS}=h(SID_{d}\left\|h\left(K\right)\right)$, and securely sends $K_{GS}$ to SD and makes $SID_{j}$ public.
- **Step 3.** On receiving the reply from the RA, SD saves the secret key $K_{GS}$ in its memory.

#### 4.2.2. Mobile User Registration

After system initialization, a mobile user *U* can be registered with the RA via secure channel.

In our scheme, we use the fuzzy extractor method for user biometric verification [26]. This step is necessary to reduce false negatives during biometric verification. A fuzzy extractor comprises of the following two procedures:

- ***Gen:*** It is a “probabilistic generation function” that computes a pair $\left(\sigma_{u},\tau_{u}\right)$ from the user biometrics information. The resultant $\sigma_{u}$ is the “biometric secret key” and $\tau_{u}$ is the “public reproduction parameter” necessary for reconstruction of $\sigma_{u}$ from $Bio_{u}^{\prime }$, a noisy biometric reading from the same user. Formally, $(\sigma_{u},\tau_{u})=$$Gen(Bio_{u})$.
- ***Rep:*** It is a “deterministic reproduction method” which constructs the original biometric secret key $\sigma_{i}$ using a noisy biometrics reading, $Bio_{u}^{\prime }$ and the public reproduction parameter $\tau_{i}$ provided the Hamming distance HD between $Bio_{u}$ and $Bio_{u}^{\prime }$ is less than or equal to a pre-defined error tolerance threshold value, say $\Delta_{t}$. Formally, $\sigma_{u}=$$Rep(Bio_{u}^{\prime },\tau_{u})$, with the restriction that $|HD\left(Bio_{u},Bio_{u}^{\prime }\right)\leq \Delta_{t}$.

The following steps are involved in this phase:

- **Step 1.***U* selects his/her identity $ID_{u}$ and securely sends $\left\{ID_{u}\right\}$ to RA.
- **Step 2.** If $ID_{u}$ is already registered, RA rejects the request. Otherwise, RA generates $R_{g},DID_{u}\in Z_{p}$ and computes $K_{UG}=h(ID_{u}\|h(R_{g}\left\|K)\right)$, and also sets TEMP=0. After that RA commits the tuple $\langle DID_{u},$$ID_{u},$$R_{g}\rangle$ to the user_data table in the gateway node GWN. RA also writes the credentials $\{K_{UG},$$DID_{u},$TEMP} to a smart card $SC_{u}$, and securely issues $SC_{u}$ to the user *U*.
- **Step 3.** After getting $SC_{u}$, *U* provides a password $PW_{u}$ and imprints biometric template $Bio_{u}$ at the sensor of a specific terminal. *U* uses the probabilistic fuzzy generator function $Gen(Bio_{u})$ to calculate the biometric secret ket $\sigma_{u}$ and a public reproduction parameter $\tau_{u}$ as $\left(\sigma_{u},\tau_{u}\right)=Gen\left(Bio_{u}\right)$. After that, *U* computes $A_{1}=DID_{u}⊕$$h(ID_{u}\|PW_{u}$$\|\sigma_{u})$, $A_{2}=h(DID_{u}$$\|ID_{u}$$\|\sigma_{u}\|PW_{u})$ and $A_{3}=K_{UG}⊕h(ID_{u}$$\|DID_{u}\|PW_{u}\|\sigma_{u})$, and replaces $K_{UG}$ and $DID_{u}$ in the smart card with $A_{1},A_{2},A_{3},\tau_{u}$. The smart card $SC_{u}$ finally contains the credentials $\left\{A_{1},A_{2},A_{3},\tau_{u},TEMP\right\}$.

**Figure 2:**
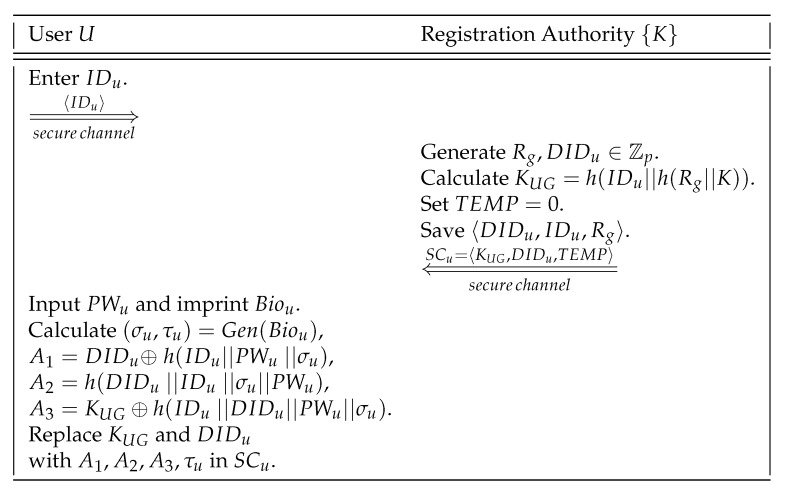
Summary of user registration.

The user registration phase is also briefed in Figure 2.

### 4.3. Login and Authentication Phase

A registered user *U* through the following steps can anonymously establish a session key with a smart device SD once mutual authentication in presence of the gateway node GWN is successful.

- **Step 1.***U* first inputs his/her identity $ID_{u}$ and password $PW_{u}$, and imprints his/her biometric $Bio_{u}$ at the sensor of a particular terminal. The smart card $SC_{u}$ of *U* then uses public $\tau_{u}$ to compute $\sigma_{u}$ from $Bio_{u}$ as $\sigma_{u}=$$Rep(Bio_{u},\tau_{u})$, and proceeds to calculate $DID_{u}=A_{1}⊕$$h(ID_{u}\|PW_{u}$$\|\sigma_{u})$ and $A_{2}^{*}=h(DID_{u}\|ID_{u}\|\sigma_{u}\|PW_{u})$. If the condition $A_{2}^{*}=A_{2}$ holds, the login is treated as successful one. In case of a failed login attempt, the smart card $SC_{u}$ increments TEMP and aborts the phase. On the other side, if it exceeds a pre-defined threshold, the user *U* is considered as compromised, and is suspended till he/she re-registers.
  After a successful login, $SC_{u}$ generates two random numbers $R_{1}$ and $w\in Z_{p}^{*}$, and calculates $K_{UG}=A_{3}⊕h(ID_{u}\|DID_{u}\|PW_{u}\|\sigma_{u})$, $M_{1}=(R_{u}\|SID_{d})⊕K_{UG}$ and $V_{1}=h(ID_{u}\|R_{u}\|K_{UG}\|M_{1})$, and dispatched the login request message $\langle DID_{u},M_{1},V_{1}\rangle$ to the GWN via public channel.
- **Step 2.** After receiving the login request $\langle DID_{u},M_{1},V_{1}\rangle$, the GWN looks up $ID_{u},R_{g}$ using $DID_{u}$ from its user_data table, and computes $K_{UG}=h(ID_{u}\|h(R_{g}\left\|K)\right)$, $(R_{u}\|SID_{d})=M_{1}⊕K_{UG}$. If $R_{u}$ is fresh, the GWN calculates $V_{1}^{*}$$=h(ID_{u}\|R_{u}\|K_{UG}\|M_{1})$. Now, if $V_{1}^{*}\neq V_{1}$, the request is considered as invalid, and the process is aborted instantly. Otherwise, the GWN generates a new random number $R_{g}^{\prime }\in Z_{p}^{*}$ and calculates $K_{GS}=h(SID_{d}\left\|h\left(K\right)\right)$, $C_{1}=h(R_{g}^{\prime }\left\|K\right)$, $C_{2}=h(ID_{u}\|R_{u}\|C_{1})$, $M_{2}=C_{2}⊕K_{GS}$ and $V_{2}=h(C_{2}\|K_{GS})$. Finally, GWN dispatches the authentication request message $\langle M_{2}$, $V_{2}\rangle$ to the accessed smart device SD via open channel.
- **Step 3.** On receiving the message $\langle M_{2}$, $V_{2}\rangle$, SD calculates $C_{2}=M_{2}⊕K_{GS}$. If $C_{2}$ is fresh, SD calculates $V_{2}^{*}=h(C_{2}\|K_{GS})$. If $V_{2}^{*}\neq V_{2}$, the request is considered as failed, and it is then aborted. On the other side, SD picks a random number $R_{d}\in Z_{p}^{*}$, computes the session key $SK=h(C_{2}\|R_{d}\|SID_{d})$ shared with *U*, $M_{3}=(R_{d}\left\|h\left(SK\right)\right)⊕K_{GS}$ and $V_{3}=h(R_{d}\|K_{GS}\left\|h\left(SK\right)\right)$. Next, SD transmits the authentication reply message $\langle M_{3},V_{3}\rangle$ to GWN via public channel.
- **Step 4.** On receiving the message $\langle M_{3},V_{3}\rangle$ from SD, GWN computes $(R_{d}\left\|h\left(SK\right)\right)=M3⊕K_{GS}$. If $R_{d}$ is also fresh, the GWN continues to calculate $V_{3}^{*}=h(R_{d}\|K_{GS}\left\|h\left(SK\right)\right)$. If $V_{3}^{*}\neq V_{3}$, the request is considered as invalid and the process is aborted immediately. Otherwise, the GWN generates another random number $DID_{u}^{\prime }\in Z_{p}^{*}$ and computes $M_{4}=(DID_{u}^{\prime }\|C_{1}\|R_{d})⊕K_{UG}$, $K_{UG}^{\prime }=h(ID_{u}\|C_{1})$ and $V_{4}=h(DID_{u}^{\prime }\|C_{1}\|R_{d}\|K_{UG}^{\prime })$. GWN then updates the tuple $\langle DID_{u}^{\prime },ID_{u},R_{g}^{\prime }\rangle$ in its user_data table, and sends the ackowledgement message $\langle M_{4},V_{4}\rangle$ to the *U* via open channel.
- **Step 5.** On receiving the message $\langle M_{4},V_{4}\rangle$ from GWN, the user *U* recovers $(DID_{u}^{\prime }\|C_{1}\|R_{d})=M4⊕K_{UG}$, and then computes $K_{UG}^{\prime }=h(ID_{u}\|C_{1})$ and $V_{4}^{*}=h(DID_{u}^{\prime }\|C_{1}\|R_{d}\|K_{UG}^{\prime })$. If $V_{4}^{*}\neq V_{4}$, the login is considered as failed one and it is aborted immediately. Otherwise, the user *U* computes the session key $SK=h(h(ID_{u}\|R_{u}\|C_{1})\|R_{d}\|SID_{d})$ and the updated values for $A_{1}^{\prime }=(R_{u}\|DID_{u}^{\prime })⊕$$h(ID_{u}\|PW_{u}$$\|\sigma_{u})$, $A_{2}^{\prime }=h(DID_{u}$$\|\sigma_{u}\|PW_{u})$, $A_{3}^{\prime }=K_{UG}^{\prime }⊕h(DID_{u}\|PW_{u}\|\sigma_{u})$. Finally, *U* resets TEMP to 0 as $TEMP^{\prime }=0$, and updates the smart card $SC_{u}$ with the values $\left\{A_{1}^{\prime },A_{2}^{\prime },A_{3}^{\prime },TEMP^{\prime }\right\}$ by replacing the old values $\left\{A_{1},A_{2},A_{3},TEMP\right\}$.

**Figure 3:**
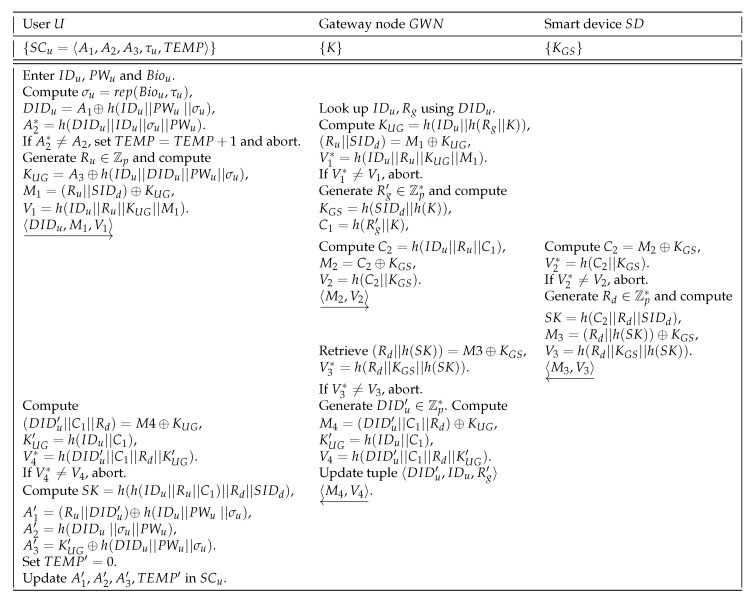
Summary of login and authentication phase.

The login and authentication phase is finally briefed in Figure 3.

**Remark** **1.** *An adversary might block the message $\langle M_{4},V_{4}\rangle$ during the communication happen in the login and authentication phase. As $DID_{u}$ and $R_{g}$ have already been updated on the gateway node GWN, the subsequent login attempts by the user U will fail. This attack can be prevented, if the gateway node GWN also maintains the old values of $DID_{u}$ and $R_{g}$ until the next successful authentication happens.*

### 4.4. Password and Biometric Update Phase

To update “password and/or biometric”, a registered user *U* inputs identity $ID_{u}$ along with the existing password $PW_{i}u$ and imprints biometric $Bio_{u}$, and then logins with the steps similar to that described in the “login and authentication phase” discussed in Section 4.3.

If the login is successful, *U* provides new password $PW_{u}^{\prime }$, imprints new biometric $Bio_{u}^{\prime }$ and recalculates $\left(\sigma_{u}^{\prime },\tau_{u}^{\prime }\right)=Gen\left(Bio_{u}^{\prime }\right)$. Next, *U* computes $A_{1}^{\prime }=DID_{u}⊕$$h(ID_{u}\|PW_{u}^{\prime }$$\|\sigma_{u}^{\prime })$, $A_{2}^{\prime }=h(DID_{u}$$\|ID_{u}$$\|\sigma_{u}^{\prime }\|PW_{u}^{\prime })$ and $A_{3}^{\prime }=K_{UG}⊕h(ID_{u}$$\|DID_{u}\|PW_{u}^{\prime }\|\sigma_{u}^{\prime })$, and replace $\left\{A_{1},A_{2},A_{3},\tau_{u}\right\}$ in the smart card $SC_{u}$ with $\left\{A_{1}^{\prime },A_{2}^{\prime },A_{3}^{\prime },\tau_{u}^{\prime }\right\}$. $SC_{u}$ now contains the updated credentials $\left\{A_{1}^{\prime },A_{2}^{\prime },A_{3}^{\prime },\tau_{u}^{\prime },TEMP\right\}$.

### 4.5. Smart Card Revocation Phase

A “lost or stolen smart card” can be revoked by requesting for a new smart card by a registered authorized user *U* to the registration authority RA via secure channel. Hence, the steps are identical to those for the mobile user registration phase as discussed in Section 4.2.2.

## 5. Security Analysis

In this section, through the widely accepted “Real-Or-Random (ROR) model” [27], the formal security analysis of the proposed scheme is presented. Furthermore, through the formal security verification tool, called AVISPA [28], the proposed scheme’s resistance to “man-in-the-middle and replay attacks” is verified. In addition, a through informal (non-mathematical) analysis presented in Section 5.3 demonstrates the proposed scheme’s resistance to various other known attacks.

### 5.1. Formal Security Analysis through Real-Or-Random Model

The ROR model proposed in [27] is widely accepted for security analysis of authentication and key agreement schemes. We describe the ROR model and then utilize the same to analyze the proposed scheme formally.

- **Participants:** Let the oracles $\pi_{U}^{u}$, $\pi_{SD}^{d}$ and $\pi_{GWN}^{g}$ denote the *u*th, *d*th and *g*th instances of a user *U*, a smart device SD and the gateway node GWN, respectively.
- **Partnering:** Two oracles $\pi_{U}^{u}$ and $\pi_{SD}^{d}$ are said to be partnered provided they share the same communication session-id sid, and the partial transcript of the exchanged messages is unique.
- **Freshness:**$\pi_{U}^{u}$ and $\pi_{SD}^{d}$ are considered fresh as long as the session key SK between *U* and SD remains unexposed to an adversary A.
- **Adversary:** The ROR model defines the DY adversary A. Formally, the adversary A can execute the queries described below.
  – $Execute(\pi^{u},\pi^{d})$: This query is modeled as an eavesdropping attack. Therefore, this query allows A to intercept the messages exchanged among *U*, SD, and GWN.
  – $Send(\pi^{d},m)$: This query is modeled an active attack. It allows A to transmit a message, say msg to an oracle $\pi^{d}$, and receive the response message in reply.
  – $CorruptSC(\pi^{u})$: Through this query, A can learn the confidential values $\{A_{1}$, $A_{2}t$, $A_{3},$$\tau_{u},$TEMP} from a user *U*’s smart card $SC_{u}$.
  – $CorruptSD(\pi^{d})$: Through this query, A can learn the secret key $\left\{K_{GS}\right\}$ stored in the captured smart device SD. The queries CorruptSC and CorruptSD are assumed to be under a weak corruption model [29] and they can not corrupt the ephemeral keys and states of the participating oracle.
  – $Test(\pi^{u},\pi^{d})$: As per the “indistinguishability in the ROR model” [27], the semantic security of the session key SK between *U* and SD can be determined by this query. To initiate, A tosses an “unbiased coin” whose outcome, say *c*, determines the output of the Test query. If SK is fresh, the oracle $\pi^{u}$ or $\pi^{d}$ produces SK, if c=1. Otherwise, if c=0, the oracle produces a random number. In all other cases, the returned value will be null.
- **Semantic security of the session key:** As per the ROR model, to compromise the semantic security of the session key, A must be able to differentiate an instance’s actual session key from a random key. A can perform a limited number of $CorruptSC(\pi^{u})$ and $CorruptSD(\pi^{d})$ queries, but can execute as many Test(·) queries as desired.
  If $Adv_{PS,A}\left(t\right)$ represents the advantage of A in compromising the semantic security of the proposed scheme PS, we have, $Adv_{PS,A}\left(t\right)=$|2.Pr[SCS]−1|, where SCS is an event of A’s success.
- **Random oracle:** All participating entities including A can invoke the “cryptographic one-way hash function”, h(·), which is further modeled as a random oracle, say HO.

Accounting to Wang et al.’s important findings [30] regarding the Zipf’s law on passwords, Theorem 1 defines the “semantic security of the proposed scheme”.

**Theorem** **1.** *Let a polynomial time adversary A attempts to break the semantic security of the proposed scheme P under the ROR model in time t. If the chosen passwords follow the Zipf’s law [30], and the bit-lengths of the biometric secret key $\sigma_{u}$ and the user identity $ID_{u}$ are $l_{1}$ and $l_{2}$, respectively, A’s advantage in compromising the semantic security of the proposed scheme PS is*

$$Adv_{PS,A}\left(t\right)\leq \frac{q_{h}^{2}}{\left|Hash\right|}+2\max\left\{C^{\prime }.q_{s}^{s^{\prime }},\frac{q_{s}}{2^{l_{1}}},\frac{q_{s}}{2^{l_{2}}}\right\},$$

*where $q_{h}$, $q_{s}$ and |Hash| represent the number of hash queries, the number of Send queries and the range of h(·), respectively, and $C^{\prime }$ and $s^{\prime }$ are the Zipf’s parameters [30].*

**Proof.**  We design our proof on the lines of the proofs that presented in [11,31,32]. Four sequential games, say $G_{i}$, i∈[0–3], are played. The event $SCS_{i}$ represents that an adversary A can successfully guess the bit *c* in the game $G_{i}$. The details regarding all the games are given below.

- **Game**$G_{0}$: This game models a real attack on the semantic security of the proposed scheme PS by A. As initially the bit *c* is guessed,
  (2) $$Adv_{PS,A}\left(t\right)=\left|2.Pr\left[SCS_{0}\right]-1\right|.$$
- **Game**$G_{1}$: This game models as an eavesdropping attack by A on PS. Through the $Execute(\pi^{u},\pi^{d})$ query, A can intercept the messages $\langle DID_{u},M_{1},V_{1}\rangle$, $\langle M_{2}$, $V_{2}\rangle$, $\langle M_{3},V_{3}\rangle$ and $\langle M_{4},V_{4}\rangle$. A can query the Test oracle and attempt to determine if the received result is the actual session key. As the session key is SK=$h(h(ID_{u}\|R_{u}\|h(R_{g}^{\prime }\left\|K))\right\|R_{d}\|SID_{d})$, and to compute the same A must learn short term secret keys ($R_{u},R_{g}^{\prime }$ and $R_{d}$) as well as long term secrets ($ID_{u},SID_{d}$ and *K*). Therefore, A gains no additional advantage for wining this game. Consequently, it follows that
  (3) $$Pr\left[SCS_{1}\right]=Pr\left[SCS_{0}\right].$$
- **Game**$G_{2}$: This game models as an active attack through use of the Send and hash queries. A attempts to beguile a legitimate entity into accepting a modified message. As discussed previously, A can repeat the queries to the oracles in order to induce hash collisions. However, since all the messages contain random nonces, hash coalitions cannot be induced on h(·) by A. It is worth noticing that both the games $G_{1}$ and $G_{2}$ are identical except for the Send and hash queries in the game $G_{2}$. Thus, through the use of birthday paradox, we have,
  (4) $$|Pr\left[SCS_{2}\right]-Pr\left[SCS_{1}\right]|\leq \frac{q_{h}^{2}}{2|Hash|}.$$
- **Game**$G_{3}$: An extension to $G_{2}$, the game $G_{3}$ is the final game and it simulates the CorruptSC and CorruptSD queries. Querying these oracles, A can learn $\{A_{1}$, $A_{2}t$, $A_{3},$$\tau_{u}$, TEMP} and $\left\{K_{GS}\right\}$, respectively. The probability of A to correctly guess the biometric secret key $\sigma_{i}$ of bit-length $l_{1}$ and the user identity $ID_{u}$ of bit-length $l_{2}$ are $\frac{1}{2^{l_{1}}}$ and $\frac{1}{2^{l_{2}}}$, respectively [33].
  As the user chosen passwords tend to follow the Zipf’s law, by utilizing trawling guessing attacks, A’s advantage will be over 0.5 when $q_{s}=10^{7}$ or $10^{8}$ [30]. If A can utilize a user’s personal information for the targeted guessing attacks, he/she will have an advantage over 0.5 when $q_{s}\leq 10^{6}$ [30]. In practical implementation, only a finite number of erroneous password attempts are permitted to the adversary A. Therefore, the games $G_{3}$ and $G_{2}$ are identical except for the guessing attacks. Thus, we can formulate the following relation as in [32]:
  (5) $$|Pr\left[SCS_{3}\right]-Pr\left[SCS_{2}\right]|\leq \max\left\{C^{\prime }.q_{s}^{s^{\prime }},\frac{q_{s}}{2^{l_{1}}},\frac{q_{s}}{2^{l_{2}}}\right\}.$$
  However, A must guess a bit $c^{\prime }$ after executing the Test query to win the game $G_{3}$. Therefore, it follows that
  (6) $$|Pr\left[SCS_{3}\right]=\frac{1}{2}.$$

From Equations (2), (3) and (6), we have,

(7) $$\begin{array}{cc} \frac{1}{2}Adv_{PS,A}\left(t\right) & =|Pr\left[SCS_{0}\right]-\frac{1}{2}|\\ & =|Pr\left[SCS_{1}\right]-\frac{1}{2}|\\ & =|Pr\left[SCS_{1}\right]-\left|Pr\left[SCS_{3}\right]\right|. \end{array}$$

Summing the inequalities from Equations (4) and (5), we obtain the following relation:

(8) $$\begin{array}{c} |Pr\left[SCS_{2}\right]-|Pr\left[SCS_{1}\right]|+|Pr\left[SCS_{3}\right]-\left|Pr\left[SCS_{2}\right]\right|\\ \leq \frac{q_{h}^{2}}{2|Hash|}+\max\left\{C^{\prime }.q_{s}^{s^{\prime }},\frac{q_{s}}{2^{l_{1}}},\frac{q_{s}}{2^{l_{2}}}\right\}. \end{array}$$

Simultaneously solving Equations (7) and (8), we arrive at the desired result:

$$Adv_{PS,A}\left(t\right)\leq \frac{q_{h}^{2}}{\left|Hash\right|}+2\max\left\{C^{\prime }.q_{s}^{s^{\prime }},\frac{q_{s}}{2^{l_{1}}},\frac{q_{s}}{2^{l_{2}}}\right\}.$$

 □

### 5.2. Formal Security Verification through AVISPA Simulation

AVISPA is an automated software tool for the formal verification of security-sensitive protocols and applications [28]. AVISPA implements the Dolev-Yao (DY) threat model and verifies whether a scheme is resistant to replay and man-in-the-middle attacks. A security protocol to be verified needs to be modeled in the associated “High Level Protocol Specification Language (HLPSL)” [34]. AVISPA provides a translator, known as HLPSL2IF, for translating HLSPL into the Intermediate Format (IF). The IF can be interpreted by one of the available four backends to generate a report in the Output Format (OF). The structure of the OF contains following:

- SUMMARY: It states if the tested protocol is “safe”, “unsafe”, or if the analysis was “inconclusive”.
- DETAILS: It reports the explanation relevant to the SUMMARY section.
- PROTOCOL: It provides the protocol to be verified.
- GOAL: It states the goal as specified in the HLPSL.
- BACKEND: It mentions the backend that has been utilized.
- STATISTICS: It provides the trace for the vulnerabilities to the target protocol, if they are present, with additional useful statistics.

A more detailed report on AVISPA and HLPSL is available at in [28]. The four backends available with AVISPA are [28]: (a) “On-the-fly Model-Checker (OFMC)”, (b) “Constraint-Logic-based Attack Searcher (CL-AtSe)”, (c) “SAT-based Model-Checker (SATMC)”, and (d) “Tree Automata based on Automatic Approximations for the Analysis of Security Protocols (TA4SP)”. Among these, OFMC and CL-AtSe are most widely accepted, and we evaluate the proposed scheme under these backends to formally verify its resistance to the “man-in-the-middle and replay attacks”.

**Figure 4:**
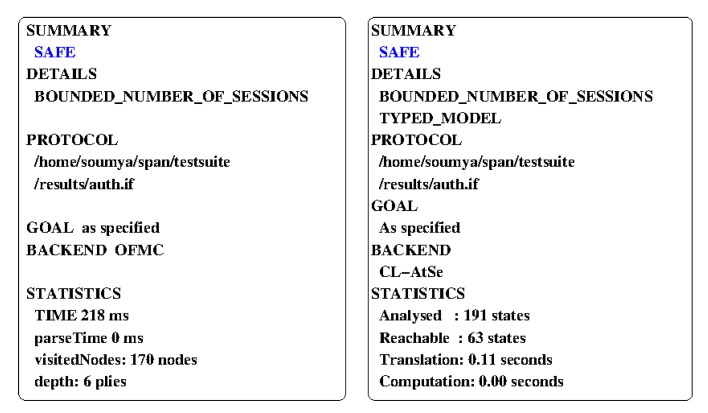
The simulation results under OFMC & CL-AtSe back-ends.

We have implemented the proposed scheme in HLSPL and defined the necessary roles for a user *U*, a smart device SD, and the GWN for the different phases of the proposed scheme. We have also specified the roles for the session, goal, and environment as per the HLPSL specification. Finally, we have simulated the proposed scheme using the “SPAN, the Security Protocol ANimator for AVISPA tool’’ [35]. Figure 4 presents the simulation results under the widely-used OFMC and CL-AtSe backends. The simulation results clearly demonstrate that the proposed scheme is safe against the “man-in-the-middle and replay attacks”.

### 5.3. Informal Security Analysis

In the following, we demonstrate that the proposed scheme is secure against various known attacks.

#### 5.3.1. Replay Attack

Assuming an adversary A replays the old message $M_{1}$ to GWN, GWN will reject the replayed message after it detects that $R_{u}$ is not fresh. Similarly, all messages are composed of random nonces, which can be further verified for their freshness. Thus, the proposed scheme is resilient against replay attack.

#### 5.3.2. Forgery Attack

An adversary A can attempt to forge the message $\langle DID_{u},M_{1},V_{1}\rangle$ to the GWN. However, $M_{1}$ is encrypted with the secret key $K_{UG}$, and $V_{1}$ is also encapsulated with $DID_{U}$ and $M_{1}$ against forgery. A cannot forge this message. Similarly, other messages cannot be forged either, and the proposed scheme is resilient against forgery attack.

#### 5.3.3. Impersonation Attack

Assuming an adversary A, after capturing the messages from a successful login an authentication attempts, to impersonate the user *U*. But, as $DID_{u}$ is of single-use and $V_{1}$ encapsulates $ID_{U}$ and $M_{1}$ against forgery, A cannot simply modify the captured messages with his/her own $R_{u}$ to impersonate *U*. Similarly, A’s attempt to impersonate the GWN will fail because he/she will be unable to generate $\langle M_{2},V_{2}\rangle$ and $\langle M_{4},V_{4}\rangle$ without the knowledge of $K_{GS}$ and $K_{UG}$, respectively. As a result, the proposed scheme is resilient against impersonation attacks.

#### 5.3.4. Man-in-the-Middle Attack

Assuming an adversary A attempts to execute a man-in-the-middle attack by capturing and modifying the login message from *U* to GWN. Nevertheless, the message cannot be forged or modified without knowledge of the secret credentials. Thus, the “man-in-the-middle attack” is also protected in the proposed scheme.

#### 5.3.5. Loss of Smart Card and Offline Guessing Attack

Assuming an adversary A recovers a lost smart card, he/she can learn the values $A_{1},A_{2},A_{3},\tau_{u}$ and TEMP through the “power analysis attacks”. Of these, except for TEMP and $\tau_{u}$, none is in plaintext and it is combination of the secret identity, password, and biometrics. It is worth noticing that $\tau_{u}$ and TEMP are the public reconstruction parameter for biometrics and failed login attempts counter, respectively, which are not sensitive. For A to subvert the proposed scheme through the offline guessing attack, he/she will have to simultaneously guess $ID_{u}$, $PW_{u}$, and $\sigma_{u}$, which is “computationally infeasible” task. Thus, the proposed scheme is resilient against the “loss of smart card and offline guessing attacks”.

#### 5.3.6. Privileged-Insider Attack

Assuming an adversary A is a privileged-insider, he/she can eavesdrop during the registration phase and learn user identity $ID_{u}$. Now, assume that he/she has subverted the user’s smart card $SC_{u}$ to recover the stored values $A_{1}=DID_{u}⊕$$h(ID_{u}\|PW_{u}$$\|\sigma_{u}),A_{2}=h(DID_{u}$$\|ID_{u}$$\|\sigma_{u}\|PW_{u})$ and $A_{3}=K_{UG}⊕h(ID_{u}\|DID_{u}\|PW_{u}\|\sigma_{u})$. It is clear that even if $ID_{u}$ is known, in order to subvert the scheme with the available information, A must simultaneously guess password $PW_{u}$ and biometric secret key $\sigma_{u}$, which is computationally infeasible. As a result, the privileged-insider attack is protected in the proposed scheme.

#### 5.3.7. Ephemeral Secret Leakage (ESL) Attack

Assume adversary A learns one or both of the session specific secrets ($R_{u}$, $R_{g},R_{d}$) through the session hijacking attack under the CK-adversary model. Since the session key $SK=h(h(ID_{u}\|R_{u}\|C_{1})\|R_{d}\|SID_{d})$ is derived from the user secret identity $ID_{u}$ and the GWN’s long term secret of *K* in addition to ($R_{u}$, $R_{g},R_{d}$), A cannot subvert the session key SK without any long term secrets. Thus, the proposed scheme is secure against ESL attack.

#### 5.3.8. Parallel Session Attack

For an adversary A to successfully execute a parallel session attack, he/she needs to compose the session key $SK=h(h(ID_{u}\|R_{u}\|C_{1})\|R_{d}\|SID_{d})$ by eavesdropping on the authentication related messages. But, no secrets are compromised regardless of lost smart card attack or privileged insider attack. As a result, the proposed scheme is secure against a parallel session attack.

#### 5.3.9. Stolen Verifier Attack

As the gateway node GWN maintains the tuple $\langle DID_{u},$$ID_{u},$$R_{g}\rangle$ for each user *U*. Of these, $DID_{u}$ and $R_{g}$ are the distict random nonces. Exposure of $ID_{u}$ is equivalent to a privileged-insider attack. However, the proposed scheme is resistant against privileged-insider attack. Thus, a stolen verifier attack is not a threat to the proposed scheme.

#### 5.3.10. Smart Card Impersonation Attack

Smart card impersonation attack can only be executed by an adversary A, if he/she can learn the secret values $ID_{u}$, $PW_{u}$ and $\sigma_{u}$ in a user’s smart card. Nevertheless, the secret values are not compromised through a lost smart card even in the presence of a privileged insider attacker. The proposed scheme is then secure against smart card impersonation attack.

#### 5.3.11. Anonymity and Untracability

Assume that an adversary A eavesdrops and monitors the messages from a successful login and authentication. None of the eavesdropped values $\left\{DID_{u},M_{1},M_{2},M_{3},M_{4},V_{1},V_{2},V_{3},V_{4}\right\}$, contains any plaintext information useful for identifying the user *U* or the smart device SD. Thus, the proposed scheme provides anonymity. Furthermore, all of the eavesdropped values are composed of some random nonces, and consequently these are always unique across different authentication sessions. Thus, the proposed scheme also provides anonymity and untracability.

## 6. Comparative Study

In this section, we benchmark the proposed scheme against the schemes proposed by Shuai et al. [7], Yu and Li [21], Naoui et al. [22], Fakroon et al. [23], and Dey and Hossain [24].

### 6.1. Communication Costs Comparison

For communication cost comparison, it is assumed that an ECC point is 320 bits, hash digest (assuming SHA-1 hashing algorithm is applied) is 160 bits, nonces as well as identities are 128 bits long. In the presented scheme, the four messages exchanged during the login and authentication phase are $\langle DID_{u},M_{1},V_{1}\rangle$ which needs (128+(128+126)+160)=544 bits; $\langle M_{2},V_{2}\rangle$ which requires (160+160)=320 bits; $\langle M_{3},V_{3}\rangle$ which demands ((128+160)+160)=448 bits and $\langle M_{4},V_{4}\rangle$ which needs ((128+160+128)+160)=576 bits. Thus, the total communication overhead of the proposed scheme turns out to be (544+320+448+576)=1888 bits =236 bytes. Table 1 summarizes the proposed scheme and other existing schemes in terms of communications overheads. From this table, we observe that the proposed scheme requires less communication overhead as compared to that for the schemes of Shuai et al. [7] and second lowest among all other schemes.

### 6.2. Computation Costs Comparison

For computation cost analysis, we denote $T_{bp},T_{m}$, $T_{b}$ and $T_{h}$ as the time needed for computing “bilinear pairing”, “ECC multiplication”, “fuzzy extractor function Gen(·)/Rep(·) for biometric verification” and “hashing” operations, respectively. Based on experimental results reported in [36], we have $T_{bp}\approx 32.713$ ms (milliseconds), $T_{m}\approx 13.405$ ms, $T_{b}\approx T_{m}=13.405$ ms and $T_{h}\approx 0.056$ ms, respectively. Table 2 briefs the computational costs for the proposed scheme and other existing schemes. It is clear that the presented scheme has a significantly less computation cost as compared to that for the schemes of Shuai et al. [7]. With the exception of Fakroon et al. [23], which might incur a greater computation cost, the proposed scheme has the lowest computation cost.

### 6.3. Security and Functionality Features Comparison

Finally, in Table 3, the functionality of the proposed scheme and other existing schemes are compared. From this table, it is apparent that the proposed scheme provides better security and functionality features features as compared to those for other existing schemes. Moreover, from the Table 1 and Table 2, we can see that the proposed scheme requires less computation and communication overheads as compared to other schemes.

## 7. Practical Impact Study through NS3 Simulation

To estimate the practicability of the proposed scheme, we have performed a simulation study. We have utilized the most recent iteration of the widely accepted network simulator tool, NS3 (3.28). We run our simulation on a Linux workstation. For our simulation, we specify the location of the gateway node (GWN) at the origin of the coordinate system. The smart devices are considered at random positions 20 to 100 m from the GWN. The users are permitted to move across a square of 150 m side centered around the gateway GWN with a maximum speed of 3 m per second. Users attempt to establish session keys with all available devices. Communication is measured across the IEEE 802.11 2.4 GHz channel. We have then simulated several scenarios with differing number of users and smart devices. The details regarding the simulation parameters are presented in Table 4. Any parameters that are not explicitly mentioned here are assumed to have their default values as defined by the NS3.

**Figure 5:**
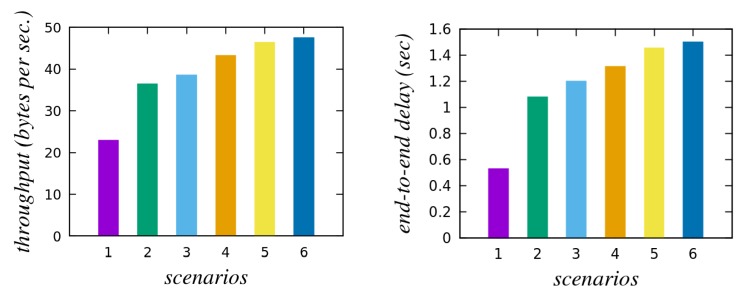
(**a**) Throughput (bytes per second) (**b**) End-to-end delay (seconds).

Figure 5a,b presents the network throughput and end-to-end delay for the proposed scheme, respectively, under different scenarios. The network throughput is calculated according to the formula:

$$\frac{N_{p}*\left|byte\right|}{T_{sum}},$$

whereas the end-to-end delay is computed with the formula:

$$\frac{\sum_{i=0}^{N_{p}}\left(T_{r_{i}}-T_{s_{i}}\right)}{N_{p}}.$$

Here, $N_{p}$ is the total number of packets received, |byte| is the number of bytes in each packet, $T_{sum}$ represents the total time taken, and $T_{s_{i}}$ and $T_{r_{i}}$ are the transmission and receiving time of the *i*th packet, respectively. The simulation results demonstrate the expected correlation between the number of participants, the network throughput and also the end-to-end delay.

## 8. Conclusions

We first discussed the issue of anonymous user authentication in smart home environments. We then cryptanalyzed the recently proposed user authentication scheme and discovered its several security vulnerabilities. Furthermore, we proposed a more secure and robust authentication scheme for anonymous user authentication and key agreement in smart homes to erase the security pitfalls found in the existing Shuai et al.’s scheme, while retaining its advantages at the same time. The security analysis and performance comparison show that the proposed scheme can provide better security and more functionality features at low communication and computation overheads, when compared these with other recent existing schemes. In our future work, we plan to investigate the possibility of extending the proposed scheme to support remote registration as it is designed in the scheme proposed by Yu and Li [21] at a more acceptable communication and computation overheads.

## Figures and Tables

**Table 1:** Communication costs comparison.

Scheme	No. of Bytes	No. of Messages
Shuai et al. [7]	(108 + 84 + 36 + 68) = 296	4
Yu and Li [21]	(84 + 124 + 164 + 164) × 2 = 1072	8
Naoui et al. [22]	(104 + 52 + 56 ) = 212	3
Fakroon et al. [23]	(100 + 52 + 52 +84) = 288	4
Dey and Hossain [24]	(132 + 132 + 52 + 52 + 52) = 420	5
Proposed scheme	(68 + 40 +56 +72) = 236	4

**Table 2:** Computation costs comparison.

Scheme	*U*	GWN	SD	Total Cost
Shuai et al. [7]	$6T_{h}+1T_{m}$	$7T_{h}+1T_{m}$	$3T_{h}$	$16T_{h}+3T_{m}$
	≈ 13.741 ms	≈ 13.797 ms	≈ 0.168 ms	≈ 27.604 ms
Yu and Li [21]	$7T_{h}+14T_{m}$	$12T_{h}+19T_{m}+4T_{bp}$	$7T_{h}+14T_{m}$	$26T_{h}+47T_{m}+4T_{bp}$
	≈ 188.062 ms	≈ 386.219 ms	≈ 188.062 ms	≈762.343 ms
Naoui et al. [22]	$12T_{h}+3T_{sym}+2T_{m}$	$13T_{h}+4T_{sym}+2T_{m}$	$1T_{h}+1T_{sym}$	$26T_{h}+7T_{sym}+4T_{m}$
	≈ 32.453 ms	≈ 34.166 ms	≈ 1.713 ms	≈68.332 ms
Fakroon et al. [23]	$4T_{h}$	$5T_{h}$	$24T_{h}$	$33T_{h}$
	≈ 0.224 ms	≈ 0.28 ms	≈ 1.344 ms	≈1.848 ms
Dey and Hossain [24]	4Th+2Tm+3Tsym	-	3Th+2Tm+3Tsym	7Th+4Te+6Tsym
	≈ 32.005 ms	≈ 0.0 ms	≈ 31.949 ms	≈63.954 ms
Proposed	$10T_{h}+1T_{b}$	$10T_{h}$	$4T_{h}$	$24T_{h}+1T_{b}$
	≈ 13.965 ms	≈ 0.56 ms	≈ 0.224 ms	≈14.749 ms

**Table 3:** Security & functionality features comparison.

Feature	$V_{1}$	$V_{2}$	$V_{3}$	$V_{4}$	$V_{5}$	$V_{6}$	$V_{7}$	$V_{8}$	$V_{9}$	$V_{10}$	$V_{11}$
Shuai et al. [7]	☑	☑	☑	☑	☑	☒	☒	☒	☒	☒	☒
Yu and Li [21]	☑	☑	☑	☑	☑	☑	☑	☑	☑	☑	☑
Naoui et al. [22]	☑	☑	☑	☑	☑	☒	☒	☒	☒	☒	☒
Fakroon et al. [23]	☑	☑	☑	☑	☑	☒	☒	☒	☑	☑	☒
Dey and Hossain [24]	☒	☒	☒	☑	☒	☒	NA	NA	☒	NA	☒
Proposed	☑	☑	☑	☑	☑	☑	☑	☑	☑	☑	☑

*Note:* ☑: The scheme is resilient against an attack or it supports a feature; ☒: The scheme is not secure against an attack or it does not support a feature; ⧆: Discussed in text. $V_{1}$: “user anonymity”, $V_{2}$: “sensor node anonymity”, $V_{3}$: “untraceability”, $V_{4}$: “resilience against replay attack”, $V_{5}$: “resilience against man-in-the-middle attack”, $V_{6}$: “resilience against ESL attack under the CK-adversary model”, $V_{7}$: “resist off-line password guessing attack”, $V_{8}$: “resist smart card impersonation attack”, $V_{9}$: “resist parallel session attack”, $V_{10}$: “resist password change attack”, $V_{11}$: “support three-factor authentication”.

**Table 4:** Simulation parameters.

Parameter	Description	
Platform	NS3(3.28)/Ubuntu 16.04 LTS	
Network scenarios	No. of users	No. of smart devices
1	3	5
2	3	10
3	3	15
4	5	15
5	5	20
6	8	20
Mobility	Random (0–3 m/s)	
Simulation time	1200 s	
